# Homologous COVID-19 BNT162b2 mRNA Vaccination at a German Tertiary Care University Hospital: A Survey-Based Analysis of Reactogenicity, Safety, and Inability to Work among Healthcare Workers

**DOI:** 10.3390/vaccines10050650

**Published:** 2022-04-20

**Authors:** Valentin Niekrens, Jan Esse, Jürgen Held, Carina Sophia Knobloch, Philipp Steininger, Bernd Kunz, Christof Seggewies, Christian Bogdan

**Affiliations:** 1 Mikrobiologisches Institut-Klinische Mikrobiologie, Immunologie und Hygiene, Universitätsklinikum Erlangen and Friedrich-Alexander-Universität (FAU) Erlangen-Nürnberg, Wasserturmstraße 3/5, D-91054 Erlangen, Germany; valentin.niekrens@extern.uk-erlangen.de (V.N.); jan.esse@uk-erlangen.de (J.E.); juergen.held@uk-erlangen.de (J.H.); bernd.kunz@uk-erlangen.de (B.K.); 2 Occupational Health Office, Institute and Outpatient Clinic of Occupational, Social and Environmental Medicine, Friedrich-Alexander-Universität (FAU) Erlangen-Nürnberg, Henkestraße 9–11, D-91054 Erlangen, Germany; carina.knobloch@fau.de; 3 Virologisches Institut-Klinische und Molekulare Virologie, Universitätsklinikum Erlangen and Friedrich-Alexander-Universität (FAU) Erlangen-Nürnberg, Schlossgarten 4, D-91054 Erlangen, Germany; philipp.steininger@uk-erlangen.de; 4 Medical Informatics and Communication Center, Universitätsklinikum Erlangen, Glückstraße 11, D-91054 Erlangen, Germany; christof.seggewies@uk-erlangen.de; 5 Medical Immunology Campus Erlangen, Friedrich-Alexander-Universität (FAU) Erlangen-Nürnberg, Schlossplatz 1, D-91054 Erlangen, Germany

**Keywords:** COVID-19, SARS-CoV-2, mRNA vaccination, BNT162b2, Comirnaty^®^, reactogenicity, incapacity to work, online survey

## Abstract

At the start of the SARS-CoV-2 pandemic, healthcare workers had an increased risk of acquiring coronavirus disease (COVID)-19. As tertiary care hospitals are critical for the treatment of severely ill patients, the University Hospital Erlangen offered BNT162b2 mRNA vaccination against COVID-19 to all employees when the vaccine became available in Germany. Here, we performed a survey to assess the age- and sex-dependent reactogenicity and safety of BNT162b2 in a real-life setting with a special emphasis on the rate of vaccine-related incapacity to work amongst the employees. All vaccinated employees were invited to participate in the survey and received access to an electronic questionnaire between 31 March and 14 June 2021, which allowed them to report local and systemic adverse effects after the first or second vaccine dose. A total of 2372 employees completed the survey. After both the first and second dose, women had a higher risk than men for vaccine-related systemic side effects (odds ratio (OR) 1.48 (1.24–1.77) and 1.49 (1.23–1.81), respectively) and for inability to work (OR 1.63 (1.14–2.34) and 1.85 (1.52–2.25), respectively). Compared to employees ≥ 56 years of age, younger vaccinated participants had a higher risk of systemic reactions after the first (OR 1.35 (1.07–1.70)) and second vaccination (OR 2.08 (1.64–2.63)) and were more often unable to work after dose 2 (OR 2.20 (1.67–2.88)). We also recorded four anaphylactic reactions and received two reports of severe adverse effects indicative of vaccine complications. After the first and second vaccination, 7.9% and 34.7% of the survey participants, respectively, were temporarily unable to work, which added up to 1700 days of sick leave in this cohort. These real-life data extend previous results on the reactogenicity and safety of BNT162b2. Loss of working time due to vaccine-related adverse effects was substantial, but was outweighed by the potential benefit of prevented cases of COVID-19.

## 1. Introduction

At the end of 2019, a new respiratory disease emerged from Wuhan in the Chinese province of Hubei. On 9 January 2020, the infectious agent was identified as a novel coronavirus, called severe acute respiratory syndrome coronavirus 2 (SARS-CoV-2). The WHO declared a state of public health emergency of international concern on 30 January 2020 and named the resulting disease coronavirus disease 2019 (COVID-19) on 11 February 2020 [1]. As of 18 February 2022, over 418 million infections and more than 5.8 million deaths from 223 countries have been recorded worldwide [2]. To combat this new infectious disease threat to humans, SARS-CoV-2 vaccines were rapidly developed using different technologies. So far, these approaches have led to the authorization of five different vaccines by the European Medical Agency (EMA), which, in Germany, are recommended to be used by the Standing Vaccination Committee (STIKO) at the Robert Koch Institute. The currently available vaccines either contain mRNA coding for the SARS-CoV-2 spike protein of the viral envelope (BNT162b2 (Comirnaty^®^) by BioNTech/Pfizer (Mainz, Germany), authorized on 21 December 2020; mRNA-1273 (Spikevax^®^) by Moderna (Cambridge, MA, USA), authorized on 6 January 2021), consist of an adenoviral vector that carries the gene of the SARS-CoV-2 spike protein (ChAdOx1/AZD1222 (Vaxcevria^®^) by AstraZeneca (Cambridge, UK), authorized on 29 January 2021; Ad26.COV2.S/COVID-19 Vaccine Janssen by Johnson&Johnson (New Brunswick, NJ, USA), authorized on 11 March 2021) or are comprised of recombinantly produced SARS-CoV-2-spike protein and Matrix M^TM^ as adjuvant (NVX-CoV2373 (Nuvaxovid^®^) by Novavax (Gaithersburg, MD, USA), authorized on 20 December 2021) [3,4].

In Germany, the COVID-19 vaccination campaign began, after the STIKO had published its first recommendation for vaccination against COVID-19 on 17 December 2020 [5]. Based on the analysis of risks for infection, severe disease, hospitalization, and/or death, the STIKO defined people over the age of 80, residents of elderly or nursing homes, and healthcare personnel with direct patient contact and very high risk of SARS-CoV-2-exposure as priority group 1 for COVID-19 vaccination [5]. Since then, 75.1% of the German population have been fully vaccinated against COVID-19 (status of 18 February 2022) [6]. Comirnaty^®^ by BioNTech/Pfizer makes up more than 71% of all vaccine doses applied in Germany [7].

At the beginning of the SARS-CoV-2 pandemic, hospital and healthcare workers had an increased risk of acquiring coronavirus disease (COVID)-19 [8]. Thus, at the University Hospital of Erlangen (UKER), a tertiary-care hospital in the Federal State of Bavaria in Germany, the vaccination of employees started on 27 December 2020, immediately after the first COVID-19 vaccine became available. The COVID-19 task force of UKER established a ranking according to the recommendations of the STIKO [5] that allowed prioritizing the vaccination of groups of hospital staff according to their occupational risk for SARS-CoV-2 infections as long as the supply with COVID-19 vaccines was limited. The only vaccine used at UKER was BNT162b2 (Comirnaty^®^; manufactured and distributed by BioNTech/Pfizer).

Considering that—at the time of authorization of BNT162b2 by the EMA—our knowledge on the safety of this new vaccine was essentially restricted to the published data of the clinical trials [9,10] and the EMA assessment report [11], continuous post-marketing studies are crucial, in particular with respect to aspects that have not yet been analyzed. We therefore decided to assess the frequency, severity, and duration of adverse effects following vaccination of the UKER hospital staff with BNT162b2, with a special emphasis given to the occupational consequences of the vaccination campaign.

## 2. Methods

### 2.1. Setting

Following the start of the COVID-19 vaccination campaign at UKER on 27 December 2020, all employees had received an offer for vaccination by 3 March 2021. The hospital vaccination campaign was announced on the intranet homepage along with information material, regularly updated answers to frequently asked questions, and the necessary documents for informed consent. The last appointments for receiving the first or second vaccine dose were made on 23 April 2021. By 26 May 2021, 7656 and 7447 UKER employees had been vaccinated once or twice, respectively. Reasons for missing the second dose included intermittent infections, severe reactions to the first vaccine dose, or diagnosis of pregnancy between the first and second vaccination. The vaccinations were carried out in the COVID-19 vaccination center of the University Hospital that was newly established in one of the lecture halls on the University Hospital campus and run by medical doctors, medical assistants, and administrative personnel. Sixteen UKER employees per day, including one emergency physician at any given time, spent a total of 8042 h of work at 91 days in the vaccination center. Following vaccination, the vaccinated participants were routinely observed for at least 15 min to recognize potential severe adverse allergic effects.

### 2.2. Study Design and Participants

To assess vaccination-related adverse effects, an online survey in German language (for an English translation see Supplementary Text S1) was developed and subsequently approved by the hospital board, the data protection officer, the staff council, and the department for quality management. The survey was made available through the UKER intranet on 31 March 2021. All vaccinated employees of UKER were electronically invited to participate in the survey via a message sent to their business e-mail addresses. In May 2021, a reminder was sent to increase the response rate. Processing of the questionnaire was possible until 14 June 2021. Due to the primarily observational character of the survey, no sample size was calculated. The evaluation was structured in accordance with the STROBE guidelines for cross-sectional studies [12] (Supplementary Table S1).

### 2.3. Instrument

The survey on COVID-19 vaccination-related adverse effects collected demographic and medical data—such as age, sex, height, weight, and medical history—to identify at-risk groups. We also enquired about the dates of vaccination, the prophylactic or therapeutic use of medication to combat side effects, the occurrence of a SARS-CoV-2 infection before, after, or between the two vaccinations and the experienced local and systemic adverse effects. The following adverse effects were included in the survey: reactions at the injection site (pain, swelling, redness, itch, rash, urticaria, other), reactions of the integument and lymph nodes (rash, itch, urticaria, swelling of lymph nodes, other), systemic reactions (chills, malaise, nausea, vomiting, fatigue, other), pain (headache, limb pain, arthralgia, myalgia, other), feverishness and the measured body temperature, anaphylaxis, facial paralysis and other neurological side effects, and the inability to work. The participants were asked about the occurrence, the duration (date of start and end), and the severity of these symptoms (scale from 1 (very mild) to 10 (very severe)) after the first and second vaccine dose. Minor incomplete answering of questions of the survey was not a criterion for exclusion from the analyses.

### 2.4. Analyses

*Statistical methods.* The collected data were anonymized and evaluated using SPSS (version 24, IBM, Armonk, NY, USA). To determine differences between groups, odds ratios (OR) with 95% confidence intervals (95% CI) were calculated. To check possible inter-correlations among relevant predictors, a logistic regression analysis was executed. The results of this bivariate analysis are given as adjusted odds ratios (aOR) with 95% confidence intervals (95% CI). Significant differences were assessed via chi-squared tests for dichotomous variables.

*Preservation of working time due to COVID-19 vaccination.* To estimate the possible preservation of working time following COVID-19 vaccination at the UKER, we had to subtract the days of sick leave due to vaccination-related adverse effects from the days of sick leave due to symptomatic SARS-CoV-2 infection. Sick leave after vaccination was easy to determine from the information provided in the electronic questionnaires. However, days of sick leave after SARS-CoV-2 infection are highly dependent on the incidence and thus on the time period considered. We therefore decided to use the mean incidence of the entire period from February 2020, when the first case of SARS-CoV-2 infection was diagnosed amongst UKER staff, to April 2021, when the last employee was vaccinated. During this time period, 408 UKER staff members were diagnosed with SARS-CoV-2 infection by PCR. As immediate PCR-based testing has been mandatory at UKER in case of (a) any respiratory symptoms, (b) high-risk SARS-CoV-2 exposures, and/or (c) after return from high-risk areas, and as all employees working on COVID-19 wards have been offered regular SARS-CoV-2-PCR testing, we anticipated a high capture rate, even of asymptomatic infections. This assumption is supported by the testing of 80 UKER healthcare workers for the presence of SARS-CoV-2 nucleocapsid protein antibodies, (Anti-SARS-CoV-2 NCP ELISA (IgG), EUROIMMUN Medizinische Labordiagnostika AG, Lübeck, Germany), the presence of which indicate infection but not vaccination. Only staff members with a history of known SARS-CoV-2 infection tested positive for SARS-CoV-2 nucleocapsid protein antibodies [13].

## 3. Results

### 3.1. Characteristics of the Survey Participants

**Figure 1:**
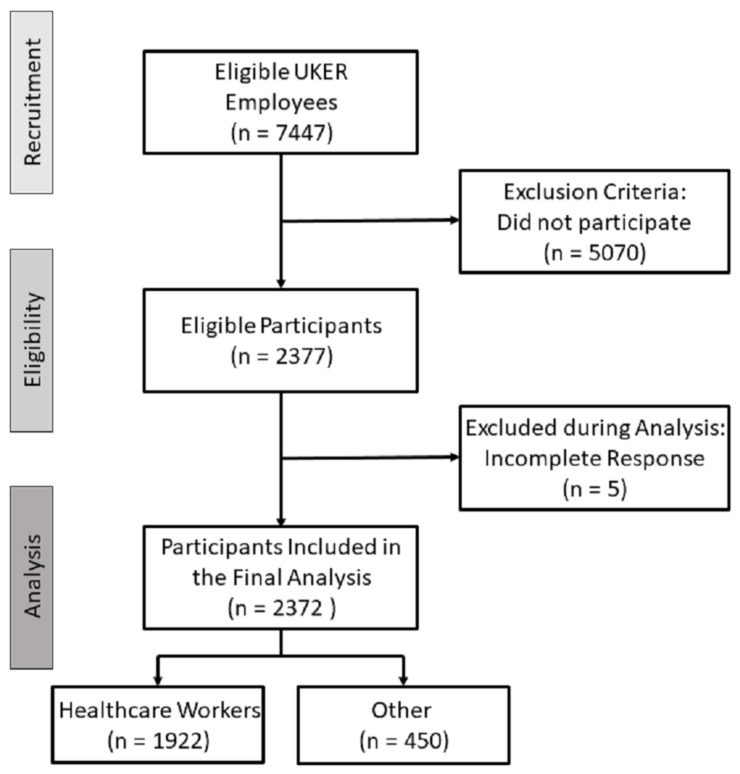
Flowchart of study participants.

A total of 2377 employees, 81% of which were healthcare professionals (nursing staff, physicians, research staff, scientists, pharmacists, laboratory staff), participated in the survey, which equals a response rate of 31.9%. Except for five responding employees, all other participants (99.8%) provided completed questionnaires (Figure 1). Their mean (± SD) age was 40 (±12.39) years, the median age was 39 years. The mean (±SD) and median body mass index (BMI) were 24.8 (±5.1) and 23.7, respectively. The largest age group (25.9%) was <30 years of age, and most participants (60.9%) had a normal body weight. Of all participants, 1655 (69.8%) were female, 715 (30.1%) were male, and 2 (0.1%) stated themselves as diverse (the latter were excluded from sex-related analyses). Of the survey participants, 33 (1.4%) reported having had a COVID-19 infection prior to COVID-19 vaccination.

Only 17 of the 2372 survey participants did not receive a second vaccination at UKER. The reasons were intermittent COVID-19 infections after the first dose (three cases), severe adverse reactions after the first vaccine dose (one case), diagnosis of pregnancy after the first dose (one case), and further non-specified reasons (11 out of the 12 participants, who did not specify a reason, had reported adverse reactions after the first vaccine dose).

Among all participants, 41.3% indicated having some form of allergy, but nobody reported an allergy to polyethylene glycol, an excipient component of the lipid nanoparticles (enclosing the BNT162b2 mRNA) that is suspected to account for severe allergic reactions in some individuals [14,15].

### 3.2. Local and Systemic Adverse Effects

**Figure 2:**
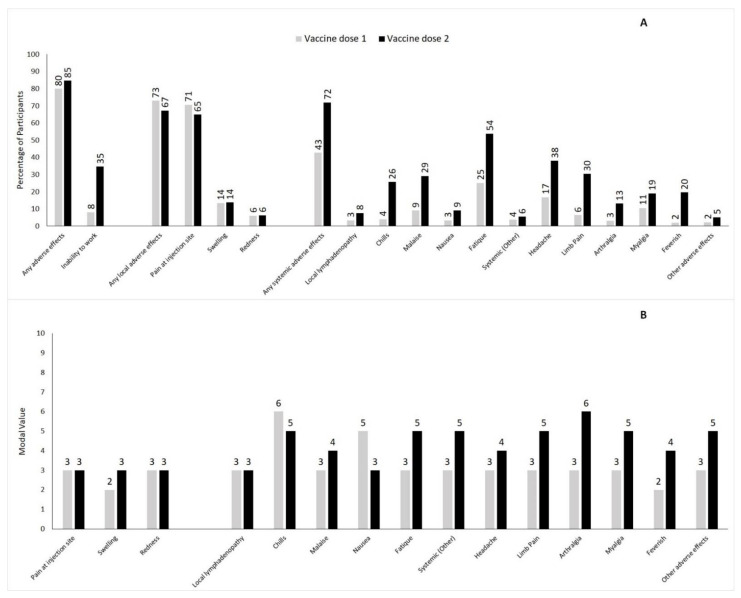
Panel (**A**) Frequency of selected local or systemic adverse effects after first and second dose of BNT162b2 mRNA vaccine given as percentage of participants. Panel (**B**) Degree of severity of selected local or systemic adverse effects after first and second dose of BNT162b2 mRNA vaccine given as modal value.

Most participants reported mild or moderate local and systemic reactions after COVID-19 vaccination. While the total frequency of systemic reactions increased from 42.8% after the first vaccine dose to 72% after the second dose, the percentage of participants reporting local reactions decreased from 73% to 67.3%. In general, all systemic adverse effects were more common after the second as compared to the first vaccination, whereas the frequency of the various local reactions either remained unaltered or decreased (Figure 2 and Supplementary Table S2). The predominant local reaction was pain at the injection site (70.7% and 64.9% after dose 1 vs. dose 2, respectively), while the most frequently occurring systemic reaction after both vaccine doses was fatigue, which was experienced by 25.1% and 53.9% of the vaccinated participants after the first or second dose, respectively. The percentage of vaccinated participants taking medication (mostly antipyretic drugs, such as ibuprofen or paracetamol) to ameliorate post-vaccination adverse effects increased from 10.9% after the first vaccination to 33% after the second vaccination.

While the intensities of local reactions were largely comparable after the first and second vaccination, the systemic reactions were more severe after the second vaccine dose (Figure 2). For instance, the modal value of severity increased for fatigue (from 3 ± 2.05 to 5 ± 2.15), arthralgia (from 3 ± 2.22 to 6 ± 2.15), and myalgia (from 3 ± 2.18 to 5 ± 2.22). Likewise, the percentage of people feeling feverish after vaccination increased from 2.1% after dose 1 to 19.7% after dose 2, and the subjective severity worsened, with the modal value for severity going up from 2 (±2.27) to 4 (±2.13). Notably, there was no significant difference between the levels of the measured body temperatures: after the first vaccine dose, 25 participants reported a temperature above 38.0°C (fever), with an average temperature of 38.9 (±0.61) °C; after the second dose, 286 participants reported fever, but the mean temperature remained unchanged (38.9 ± 0.56 °C). Thus, the measured value of the body temperature did not reflect the subjectively perceived aggravation of feeling feverish.

### 3.3. Impact of Age

Comparing the survey participants below 56 years to those 56 years or older [10], adverse effects were more frequent among those of a younger age, especially after the second dose. Significantly increased odds ratios were calculated for the swelling of lymph nodes (OR 2.53 (95% CI 1.40–4.60)), chills (OR 2.50 (95% CI 1.81–3.44)), arthralgia (OR 1.60 (95%CI 1.09–2.36)), and other adverse effects in the younger age group after the second dose (Table 1). Age boundaries were chosen in accordance with previous clinical trials [9,10]. Five survey participants forgot to report their age.

### 3.4. Impact of Sex

In females, several local or systemic adverse effects occurred significantly more often than in males after the first and/or second vaccination (Table 2). For example, after the first dose of the BNT162b2 vaccine, nausea (OR 2.78 (95% CI 1.47–5.29)) and headache (OR 1.89 (95% CI 1.45–2.46)) were more frequently reported by females than by males. After the second dose, arthralgia (OR 1.60 (95% CI 1.20–2.12)), myalgia (OR 1.60 (95% CI 1.25–2.03)), swelling of lymph nodes (OR 2.05 (95% CI 1.38–3.04)) and chills (OR 1.45 (95% CI 1.17–1.79)) occurred more often in females than in males.

### 3.5. Intercorrelation Analysis

Female sex and age below 56 years proved to be mostly independent risk factors for the occurrence of local and systemic adverse reactions after BNT162b2 vaccination (Table 3).

### 3.6. Participants with Allergies

We conducted a similar analysis of odds ratios comparing participants with and without a history of allergies. Most side effects were tentatively, but not significantly, more frequent in allergic participants, except for some local reactions such as swelling (OR 1.39 (1.10–1.76) after dose 1; OR 1.28 (1.01–1.61) after dose 2) or redness at the injection site (OR 1.46 (1.04–2.03) after dose 2).

### 3.7. Severe Adverse Events

From the 2372 participants, we received five reports of facial paralysis, two following the first and three following the second vaccine dose (range of onset from zero to three days after vaccination; one participant reported facial paralysis after the first and the second vaccine dose without reporting the date of onset). Other vaccination-related complications included severe vertigo in a 50-year-old female (starting 3 days after the first vaccine dose and persisting for several months) and facial indurations and swellings in areas of previous injections of a hyaluronic acid filler in a woman below age 40, which began one day after the first vaccination and was accompanied by prominent regional lymphadenitis.

During the 15 min post-vaccination observation period, five cases of acute severe adverse reactions were observed (Table 4), which were rated as anaphylactic or allergic reactions. The individuals received initial medical care by the emergency team present in our vaccination center and subsequently by the emergency department of UKER. Only one person was required to stay overnight in the hospital.

### 3.8. Inability to Work

After the first vaccine dose, 187 (7.9%) of the participants reported an inability to work and 166 persons gave precise information on its duration, whereas after the second dose, 817 participants (34.7%) were unable to work and 789 provided details. The mean time of absence from work due to vaccination-related side effects was 1.96 (±2.5) days after the first dose and 1.74 (±1.6) days after the second dose. The range was 1 to 26 days after the first dose and 1 to 23 days after the second vaccination. Altogether, the 2372 employees participating in the survey reported 326 days of absence from work after the first and 1374 days after the second vaccination, which on average adds up to 0.72 days of absence per fully vaccinated staff member. Those who were unable to work mostly had systemic or systemic and local reactions following vaccination; after the first and second dose, only 21 or 3 vaccinated participants, respectively, stayed away from work solely due to local reactions.

In accordance with the higher frequency of adverse effects reported by younger and female participants (see above), women were more likely to be unable to work; this was true for all age groups and after both the first (OR for women compared to men 1.63 (1.14–2.34)) and the second vaccine dose (OR 1.85 (1.52–2.25)) (Table 5).

### 3.9. Estimated Preservation of Working Time Due to COVID-19 Vaccination

If vaccination would have been available since the beginning of the COVID-19 pandemic to the complete UKER staff (*n* = 8560 in November 2020), a major proportion of the 408 SARS-CoV-2 infections detected by PCR amongst UKER employees until April 2021 could have potentially been prevented based on the reported vaccine efficacy of 95% [10]. The time of SARS-CoV-2 positivity (first positive to first negative PCR result) of UKER staff members averaged 23 days before the institutional vaccination campaign started (data collected from October to November 2020; Esse J et al., unpublished data). This is in accordance with Phillips et al. [16], who observed an average of 25 days between the first positive and two consecutive negative SARS-CoV-2 PCR tests. As SARS-CoV-2-infected UKER employees were allowed to resume work only after clinical cure and at least one negative SARS-CoV-2 PCR test, the duration of PCR positivity in days is identical to the period of sick-leave due to a SARS-CoV-2-infection. Therefore, the maximal preventable loss of working time equals 8915 days (Part 1 of Supplementary Text S2). Taking into account the 6163 days of inability to work due to vaccination-related adverse effects, vaccination would still lead to an estimated preservation of working time of 2752 days (Part 2 of Supplementary Text S2). In fact, the preservation of working time due to vaccination would already outweigh the incapacity to work due to vaccine-induced adverse effects if the number of prevented infections exceeded 282, which equals 3.3% of the UKER employees (Part 3 of Supplementary Text S2).

## 4. Discussion

In the present study, we observed that the employees of the University Hospital Erlangen, Germany, frequently developed local and systemic reactions after the first and second BNT162b2 vaccination. Younger age groups were particularly affected. The vast majority of reactions were of mild or moderate nature and self-resolving, which is largely in line with the results of the previously published phase 3 clinical trial on the BNT162b2 vaccine and other real-world studies [10,17,18,19,20,21,22]. Most importantly, our analysis revealed a preponderance of adverse effects amongst females and an unexpectedly high rate of inability to work, in particular after the second vaccine dose. Higher rates of females reporting vaccination-related adverse effects have also been observed after other vaccinations [23]. The stronger reactions amongst females may be due to hormonal, immunological or genetic/epigenetic factors or could result from differences in the microbiota between males and females, while behavioral factors may add to the higher reporting rate by females [24,25]. Furthermore, we estimated the preservation of working time that would have resulted from a theoretical start of the COVID-19 vaccination campaign at the beginning of the pandemic. Our calculations strongly suggest that the preservation of working time due to the prevention of COVID-19 by vaccination clearly outweighed the reported absence from work due to vaccine-related side effects, thus documenting the benefit of the institutional vaccination campaign.

The observed increase in frequency and severity of systemic reactions after the second vaccine dose would have likely been even more pronounced if 33% of the survey participants had not taken antipyretic drugs. On the other hand, the use of antipyretic drugs may also have been a consequence of severe side effects. Several participants reported severe adverse events or complications after receiving the vaccine. While vaccinated participants with a history of known allergies did not have a higher general risk of side effects, we noted an unexpectedly high percentage of acute anaphylactic reactions after BNT162b2 vaccination in this group. Four anaphylactic reactions in the context of 16,704 vaccinations correspond to a rate of 0.024%. This is roughly 60 times higher than the reported percentage of 0.0004% taken from the VAERS database in the USA [26], but closely resembles the rate of anaphylaxis (0.027%) observed amongst employees of the Massachusetts General Hospital in Boston following BNT162b2 vaccination [27]. The increased rate of acute, anaphylactic reactions observed at the UKER vaccination center might be due to the fact that the UKER medical personnel taking care of COVID-19 patients showed a high vaccine acceptance, even with a positive history of allergies, while in the general population people with similar medical records might be more reluctant to become vaccinated. The UKER vaccination center provided above-average medical care, with experienced emergency doctors on-site and the emergency department and intensive care unit of the UKER only minutes away, which likely encouraged persons with a history of allergic reactions to receive the vaccine and thereby contributed to the higher rate of reported anaphylactic reactions. Of note, only allergic reactions that required drug treatment were classified as vaccine-related severe adverse effects. Whether some of the recorded cases (Table 4) would have also regressed without treatment remains unclear.

We also received five reports of facial paralysis from the 2372 employees participating in the survey. Again, this appears to be an unexpectedly high number, considering the data reported in the phase 3 clinical trial [10] and the failure to detect an increased risk for facial paralysis after BNT162b2 vaccination in German [28] and US pharmacovigilance analyses [29]. Due to the anonymization of the data, we could not follow up on these cases to verify whether the symptoms were described accurately and to exclude other possible reasons for the occurrence of a facial paralysis. The same holds true for the other two cases of vaccine complications (severe and persistent vertigo, facial swelling, and induration plus regional lymphadenitis) that were reported.

Our findings underline that mRNA vaccines such as BNT162b2 require continuous monitoring with the help of spontaneous or active surveillance systems and a subsequent thorough evaluation of the reports by national institutions responsible for pharmacovigilance such as the Paul Ehrlich Institute in Germany. This necessity is further underlined by current efforts of cross-national studies on COVID-19 vaccine safety [20]. Since widespread booster vaccinations using COVID-19 mRNA vaccines have been recommended and implemented in many countries including Germany [30], it will be of particular importance to also record the frequency and severity of adverse effects following the third vaccine dose.

The highest risk for side effects and inability to work after vaccination was seen in younger employees and particularly in females (Table 5), which proved to be mostly independent risk factors (Table 3). Considering the age and sex structure of healthcare workers, it is critical to stagger vaccination appointments so that not all co-workers of one department or service unit became vaccinated at the same time. There are anecdotal reports from the beginning of the vaccination campaign in Germany and other countries, where hospital wards or units had to temporarily close down due to vaccination-related side effects in too many employees. Thus, a well-planned vaccination strategy will help to avoid a negative impact of institutional vaccination campaigns due to sick leave on patient care. Most importantly, in the long run, absence from work due to vaccine-related side effects is outweighed by the preservation of working time resulting from the vaccine-mediated prevention of COVID-19 amongst the employees. As the vaccine-related preservation of working time depends on the number of prevented infections and therefore on the incidence of COVID-19, the impact of vaccination and the estimation of preserved working time will depend on the epidemiological situation: the lower the incidence of SARS-CoV-2-infections, the longer it will take, until the prevention of COVID-19-related sick days will compensate for sick-leaves caused by vaccination. Based on our data at UKER (Esse et al., not shown), this would have taken either 11 months during the low incidence period (February to December 2020) or 3 months during the high incidence period (November 2020 to January 2021). To carry out the given calculations, we assumed that we detected a major part of SARS-CoV-2 infected UKER staff members. The cumulative incidence of PCR-proven SARS-CoV-2 infection amongst UKER nursing staff prior to the institutional vaccination campaign was 5.55% (153 detected SARS-CoV-2 infections), while during the same time period the seroprevalence in the general population was around 2% as determined by a nationwide serological analysis [31]. These results are in accordance with findings by Wagner et al. [32], who also observed a significantly higher seroprevalence amongst healthcare workers in 2020 prior to the introduction of vaccination (OR 2.26, 95%-CI: 1.53–3.28). Thus, the assumption that all SARS-CoV-2 positive staff members were indeed detected by PCR seems plausible, although we cannot exclude a small number of unreported infected staff members. We wish to emphasize that our calculations are somewhat simplified, because they do not take into account the waning of vaccine-mediated immunity [33].

As people with strong adverse effects after vaccination may have participated more often in our survey, we cannot exclude an over-reporting of side effects and an overestimation of vaccine-related sick-leave in our study. On the other hand, readmission to work in our hospital was only possible when the employee had become asymptomatic and had a negative SARS-CoV-2 PCR. Thus, a SARS-CoV-2 infection in our setting resulted in a particularly long absence from work compared to hospitals with a lower threshold for readmission.

A strength of our study is the real-world setting and the study population, which due to its medical background and affiliation provided vigorous and reliable reports. Furthermore, the data collected from the participants of the survey enabled us to identify groups at particular risk for vaccine-related side effects as well as cases of vaccine complications. We believe that sharing the questionnaire on the intranet platform of UKER, rather than on a public website or via social media, may have increased the conscientiousness and accuracy with which the survey was completed. Furthermore, the fact that a large proportion of the survey participants were healthcare professionals likely increased the accuracy of descriptions of side effects and of the personal medical history. On the other hand, we cannot exclude that the increased awareness of healthcare professionals regarding the safety of a new vaccine might have led to more reports of side effects as compared to the general population. This could explain the diverging rates of anaphylactic reactions in medical and non-medical cohorts as suspected by Kim et al. [34] and also for the differences in the rates reported for facial paralysis following BNT162b2 vaccination [29,35]. Limitations of our study are the unequal proportion of female and male participants and the response rate of only 31.9% of all vaccinated employees compared to other studies [18,19], which may have contributed to a higher percentage of reported severe adverse effects (see above), especially with a completion rate of 99.8%. Another limitation of our study is a potential recall bias, as some participants completed the survey only weeks after having been vaccinated, which might have caused an underreporting of minor side effects (e.g., mild headaches) that were forgotten or ignored. On the other hand, the frequency of side effects reported in surveys like ours could be higher than the actual frequencies in real life, because in surveys participants suffering from side effects may respond more often compared to persons who did not experience any side effects and therefore abstain from participation. However, this error is likely to be very small, because the frequency of local and systemic side effects that reflect a normal vaccine reaction was in line with the data collected in the randomized controlled phase 3 trial [10].

## 5. Conclusions

Our analysis not only highlights a sex and age preponderance of BNT162b2 vaccine-related adverse effects, but also provides new insights into the inability to work versus the preservation of working time following an institutional vaccination campaign and underscores the need for a well-planned vaccination schedule to maintain patient care. The loss of working hours due to vaccination-related adverse effects is outweighed by the preservation of working time following BNT162b2 vaccination, which protects from severe COVID-19.

## Figures and Tables

**Table 1:** Odds ratios of adverse effects after first and second dose of BNT162b2 mRNA vaccine comparing participants aged ≤ 55 years to participants aged ≥ 56 years.

Adverse Effects	Odds Ratio (95% CI) after Dose 1	Odds Ratio (95% CI) after Dose 2
Pain at injection site	2.12 (1.68–2.67) ***	2.20 (1.75–2.77) ***
Swelling	0.96 (0.69–1.33)	0.98 (0.71–1.37)
Redness	1.09 (0.67–1.76)	0.85 (0.55–1.33)
Itching	1.20 (0.57–2.55)	1.32 (0.56–3.12)
Any local reaction	1.96 (1.55–2.49) ***	2.01 (1.60–2.53) ***
Swelling of lymph nodes	3.40 (1.24–9.35) *	2.53 (1.40–4.60) **
Chills	1.52 (0.78–2.95)	2.50 (1.81–3.44) ***
Malaise	1.50 (0.96–2.35)	3.11 (2.25–4.30) ***
Nausea	1.61 (0.77–3.37)	1.90 (1.17–3.10) **
Fatigue	1.10 (0.84–1.43)	1.79 (1.42–2.25) ***
Headache	1.63 (1.15–2.30) **	2.00 (1.55–2.59) ***
Limb pain	1.14 (0.70–1.85)	1.79 (1.36–2.36) ***
Arthralgia	0.84 (0.46–1.54)	1.60 (1.09–2.36) *
Myalgia	1.22 (0.82–1.79)	1.38 (1.01–1.89) *
Feverish	1.25 (0.53–2.97)	2.02 (1.43–2.84) ***
Any systemic reaction	1.35 (1.07–1.70) *	2.08 (1.64–2.63) ***
Inability to work	1.19 (0.76–1.86)	2.20 (1.67–2.88) ***

Grey shading signifies statistically significant increased risk (*p*-value * < 0.05, ** < 0.01, *** < 0.001) for vaccinated participants < 55 years of age as compared to vaccines > 56 years.

**Table 2:** Odds ratio of adverse effects after first and second dose of BNT162b2 mRNA vaccine comparing female to male participants.

	Odds Ratio (95% CI) after Dose 1	Odds Ratio (95% CI) after Dose 2
Pain	1.21 (1.00–1.46)	1.31 (1.09–1.57) **
Swelling	1.37 (1.05–1.80) *	1.28 (0.98–1.66)
Redness	1.30 (0.88–1.92)	1.17 (0.80–1.70)
Itching	2.08 (1.08–4.00) *	10.65 (2.58–43.95) ***
Any local reaction	1.22 (1.01–1.49) *	1.39 (1.16–1.68) ***
Swelling of lymph nodes	1.20 (0.72–2.00)	2.05 (1.38–3.04) ***
Chills	1.47 (0.91–2.39)	1.45 (1.17–1.79) ***
Malaise	1.05 (0.77–1.42)	1.38 (1.13–1.69) **
Nausea	2.78 (1.47–5.29) **	1.76 (1.25–2.48) **
Fatigue	1.35 (1.10–1.67) **	1.40 (1.17–1.67) ***
Headache	1.89 (1.45–2.46) ***	1.35 (1.12–1.62) **
Limb pain	1.40 (0.95–2.05)	1.70 (1.39–2.08) ***
Arthralgia	1.14 (0.68–1.89)	1.60 (1.20–2.12) **
Myalgia	0.95 (0.72–1.27)	1.60 (1.25–2.03) ***
Feverish	3.15 (1.34–7.44) **	1.66 (1.31–2.11) ***
Any systemic reaction	1.48 (1.24–1.77) ***	1.49 (1.23–1.81) ***
Inability to work	1.63 (1.14–2.34) **	1.85 (1.52–2.25) ***

Grey shading signifies statistically significant increased risk (*p*-value * < 0.05, ** < 0.01, *** < 0.001) for female as compared to male vaccinated participants.

**Table 3:** Bivariate analysis for factors associated with higher risk for adverse effects given in crude odds ratio (cOR) and adjusted odds ratio (aOR) after first and second dose of BNT162b2 mRNA vaccine.

		Any Local Reaction		Any Systemic Reaction		Inability to Work	
		cOR (95% CI)	aOR (95% CI)	cOR (95% CI)	aOR (95% CI)	cOR (95% CI)	aOR (95% CI)
First dose	Female vs. Male	1.22 (1.01–1.49)	1.22 (1.00–1.48)	1.48 (1.24–2.34)	1.47 (1.23–1.76)	1.63 (1.14–2.34)	1.62 (1.23–2.32)
	Age ≤ 55 vs. Age ≥ 56	1.96 (1.55–1.49)	1.96 (1.54–2.48)	1.35 (1.07–1.70)	1.33 (1.05–1.69)	1.19 (0.76–1.86)	1.17 (0.75–1.82)
Second dose	Female vs. Male	1.39 (1.16–1.68)	1.39 (1.16–1.68)	1.49 (1.23–1.81)	1.49 (1.23–1.80)	1.85 (1.52–2.25)	1.85 (1.52–2.26)
	Age ≤ 55 vs. Age ≥ 56	2.01 (1.60–2.53)	2.00 (1.59–2.52)	2.08 (1.64–2.63)	2.07 (1.64–2.62)	2.20 (1.67–2.88)	2.19 (1.66–2.89)

**Table 4:** Acute complications during the 15-min on-site observation period after the first or second BNT162b2 vaccination.

	Case 1	Case 2	Case 3	Case 4	Case 5
Age group	30–39	<30	30–39	>59	30–39
Sex	Female	Female	Female	Female	Female
Vaccination	Dose 1	Dose 1	Dose 2	Dose 2	Dose 2
If dose 2: reaction to first vaccination	-	-	Generalized skin rash after 4 h	Headache, numbness of the tongue	N/A
Known allergies	Antibiotics, analgesics, tetanus toxoid vaccine	Six previous allergic events of unknown origin that required treatment	Penicillin	-	One allergic reaction of unknown origin that required treatment
Other medical history	-	Allergic Asthma	-	Rheumatism	-
Prior medication	Cetirizine (long-term medication)	-	4 mg clemastine-fumarate i.v.	-	-
Symptoms	Tingling sensation on entire body, constricted airways, tachycardia, hypotension	Erythema of the neck, malaise, breathlessness, globus sensation	Generalized itch	Nausea, headache, heat sensation, “furry sensation” in mouth	Light-headedness, strong nausea

**Table 5:** Percentage of participants unable to work for at least one day after first and second dose of BNT162b2 vaccination.

	Male		Female	
Years of Age	Dose 1	Dose 2	Dose 1	Dose 2
<30	7.3%	30.5%	10.9%	45.9%
30–39	6.0%	27.2%	9.1%	41.1%
40–49	3.1%	23.3%	6.9%	39%
50–59	6.8%	23.8%	8.2%	31.2%
>59	3.3%	13.3%	5.5%	17.8%

## Supplementary Materials

The following supporting information can be downloaded at: https://www.mdpi.com/article/10.3390/vaccines10050650/s1. Supplementary Text S1: Content of the survey distributed amongst the vaccinated UKER employees (translated into English language). Supplementary Text S2: Estimation of working time preservation (in days) at UKER due to a theoretical COVID-19 vaccination from the beginning of the pandemic based on the number of SARS-CoV-2-infections detected by PCR amongst UKER employees. Supplementary Table S1: STROBE checklist. Supplementary Table S2: Frequency of adverse effects after first and second dose of BNT162b2 mRNA vaccine given as percentage of participants.
