# Supplementary material for: Homologous COVID-19 BNT162b2 mRNA Vaccination at a German Tertiary Care University Hospital: A Survey-Based Analysis of Reactogenicity, Safety, and Inability to Work among Healthcare Workers
Source: Vaccines (Basel). 2022 Apr 20;10(5):650. doi: 10.3390/vaccines10050650 (PMC9147477; doi:10.3390/vaccines10050650)
Supplement: Supplementary file 1 [file vaccines-10-00650-s001.zip › Supplementary Table S2-JE-CB-20-4-2022.pdf]

Supplementary Table S2:

Frequency of adverse effects after the first versus the second dose of BNT162b2 mRNA vaccine (given as percentage of all survey participants)

	After vaccine dose 1	After vaccine dose 2
**Any adverse effects**	79,5%	84,7%
**Inability to work**	7,9%	34,7%
**Local adverse effects**	73,0%	67,3%
Pain at injection site	70,7%	64,9%
Swelling	13,5%	13,8%
Redness	6,1%	6,3%
Itching at injection site	2,7%	2,2%
Rash at injection site	0,1%	0,2%
Urticaria at injection site	0,1%	0,0%
Other at injection site	2,4%	1,7%
**Systemic adverse effects**	42,8%	72,0%
Skin Rash	0,4%	0,9%
Generalized Itching	0,7%	0,6%
Urticaria	0,2%	0,2%
Local lymphadenopathy	3,4%	7,6%
Skin (Other)	0,4%	0,4%
Chills	4,1%	25,7%
Malaise	9,1%	29,2%
Nausea	3,4%	9,2%
Vomiting	0,3%	1,2%
Fatique	25,1%	53,9%
Systemic (Other)	3,7%	5,5%
Headache	16,8%	38,0%
Limb Pain	6,4%	30,4%
Arthralgia	3,2%	13,2%
Myalgia	10,6%	19,1%
Pain (Other)	1,3%	2,7%
Feverish	2,1%	19,7%
Anaphylaxis	0,0%	0,0%
Anaphylactoid reactions	0,2%	0,3%
Facial paralysis	0,1%	0,1%
Nervous System (Other)	0,4%	0,9%
**Other adverse effects**	2,3%	5,1%
