# Supplementary material for: Homologous COVID-19 BNT162b2 mRNA Vaccination at a German Tertiary Care University Hospital: A Survey-Based Analysis of Reactogenicity, Safety, and Inability to Work among Healthcare Workers
Source: Vaccines (Basel). 2022 Apr 20;10(5):650. doi: 10.3390/vaccines10050650 (PMC9147477; doi:10.3390/vaccines10050650)
Supplement: Supplementary file 1 [file vaccines-10-00650-s001.zip › Supplementary Text S1-JE-CB-20-4-2022.pdf]

Supplementary Text S1:

Survey "Side effects after COVID-19 vaccination" at University Hospital Erlangen

Thank you for participating in this survey! Mandatory fields are marked by a red star*.

Part 1: General data

Age (years):*

Weight (kg):*

Height (cm):*

Sex (d/m/w):* d ☐ m ☐ w ☐

Pre-existing conditions:* no ☐ yes ☐: Which pre-existing conditions?..

Allergies:* no ☐ yes ☐:* Which allergies?..

Occupational group:* Nursing staff with direct patient contact ☐ medical staff with direct patient contact ☐ administrative staff with direct patient contact ☐ other staff with direct patient contact ☐ administrative staff without direct patient contact ☐ laboratory staff ☐ research staff (physicians, scientists, technical assistants etc.) ☐ hospital pharmacy staff ☐ technical service (incl. IT staff) ☐ cleaning staff ☐ kitchen staff.

Part 2: First dose of vaccine

General data

Vaccine applied:* Comirnaty®/BioNtech ☐ COVID-19 Vaccine Moderna® ☐ COVID-19 Vaccine AstraZeneca ☐

Batch number:*

Date of vaccination:*

Did you take any precautionary painkillers and/or antipyretics *before* vaccination?* no ☐ yes ☐

If yes, which medication did you take, for how long, and at what dose?

- Medication:
- Duration in days:
- Dose:

Did you take painkillers and/or antipyretics *after* vaccination to treat any adverse effects?* no ☐ yes ☐

If yes, what medication did you take, for how long, and at what dose?

- Medication:
- Duration in days:
- Dose:

Were you unable to work after vaccination due to adverse effects? If you had time off, would you have been unable to work? * no ☐ yes ☐

If yes, how long were you unable to work after vaccination? (whole days):

Adverse effects

Did you experience any adverse effects:* no ☐ yes ☐

Please provide the following information for any adverse effects that occurred:

(a) onset/end of adverse effect

b) Subjective severity of adverse effect (1-10, 1 = very mild 10 = very severe).

c) Restoration of health

Side effects	Onset	End	Severity	Restoration of health
**Reactions at injection site**				
Pain ☐:				
Swelling ☐:				
Redness ☐:				
Itching ☐:				
Rash ☐:				
Urticaria ☐:				
Others ☐: Which?				
**Skin reactions**				
Rash ☐:				
Itching ☐:				
Urticaria ☐:				
Swelling of lymph nodes ☐:				
Others ☐: Which?				
**Reduction of general condition**				
Chills ☐:				
Malaise ☐:				
Nausea ☐:				
Vomiting ☐:				
Weakness, fatigue☐:				
Others ☐: Which?				
**Pain**				
Headache ☐:				
Limb pain ☐:				
Arthralgia ☐:				
Myalgia ☐:				
Others ☐: Which?				
**Fever**				
Height of temperature ☐:				
**Allergic reaction**				
Anaphylaxis ☐:				
Others ☐: Which?				
**Damage to the nervous system**				
Facial paralysis ☐:				
Others ☐ Which?				
**Other adverse effects**				
Others ☐: Which?				

Part 3: Second dose of vaccine

General data

Vaccine applied:* Comirnaty®/BioNtech ☐ COVID-19 Vaccine Moderna® ☐ COVID-19 Vaccine AstraZeneca ☐

Batch number:*

Date of vaccination:*

Did you take any precautionary painkillers and/or antipyretics *before* vaccination? * no ☐ yes☐

If yes, which medication did you take, for how long, and at what dose?

- Medication:
- Duration in days:
- Dose:

Did you take painkillers and/or antipyretics *after* vaccination to treat any adverse effects?* no ☐ yes ☐

If yes, what medication did you take, for how long, and at what dose?

- Medication:
- Duration in days:
- Dose:

Were you unable to work due to vaccination side effects? If you had time off, would you have been unable to work? * no ☐ yes ☐

If yes, how long were you unable to work after vaccination? (whole days):

Adverse effects

Did you experience any adverse effect?* no ☐ yes ☐

Please provide the following information for any adverse effects that occurred:

(a) onset/end of adverse effects

b) Subjective severity of adverse effect (1-10, 1 = very mild 10 = very severe).

c) Restoration of health

Side effects	Onset	End	Severity	Restoration of health
**Reactions at injection site**				
Pain ☐:				
Swelling ☐:				
Redness ☐:				
Itching ☐:				
Rash ☐:				
Urticaria ☐:				
Others ☐: Which?				
**Skin reactions**				
Rash ☐:				
Itching ☐:				
Urticaria ☐:				
Swelling of lymph nodes ☐:				

Others ☐: Which?				
**Reduction of general condition**				
Chills ☐:				
Malaise ☐:				
Nausea ☐:				
Vomiting ☐:				
Weakness, fatigue☐:				
Others ☐: Which?				
**Pain**				
Headache ☐:				
Limb pain ☐:				
Arthralgia ☐:				
Myalgia ☐:				
Others ☐: Which?				
**Fever**				
Height of temperature ☐:				
**Allergic reaction**				
Anaphylaxis ☐:				
Others ☐: Which?				
**Damage to the nervous system**				
Facial paralysis ☐:				
Others ☐: Which?				
**Other side effects**				
Others ☐: Which?				

<u>Part 4: Further information</u>

Have you been diagnosed with SARS-CoV-2 infection after the 2nd dose of vaccination:* no ☐ yes ☐.

Positive PCR test result:

Negative PCR test result:

Was the infection accompanied by symptoms? no ☐ yes ☐

Symptoms	Onset	End	Severity	Restoration of health
What kind of symptoms?				

If you would like to share additional personal experience with your COVID-19 vaccination that goes beyond this questionnaire or if you develop a SARS-CoV-2 infection in the future, please feel free to contact us at the Institute of Clinical Microbiology, Immunology and Hygiene, either by e-mail or phone (Jan Esse, jan.esse@uk-erlangen.de, Tel. 46918; OA Dr. Bernd Kunz, bernd.kunz@uk-erlangen.de, Tel. 46902; Prof. Dr. Christian Bogdan, christian.bogdan@uk-erlangen.de, Tel. 22551).

<u>Part 5: Optional contact details</u>

Your information will be stored anonymously. The information cannot be traced back to you.

If you agree to be contacted about information you have provided, please enter your contact details below. You cannot be contacted without this information.

First Name (optional):

Second Name (optional):

Phone Number (optional):

Email Address (optional):
