# Supplementary material for: Homologous COVID-19 BNT162b2 mRNA Vaccination at a German Tertiary Care University Hospital: A Survey-Based Analysis of Reactogenicity, Safety, and Inability to Work among Healthcare Workers
Source: Vaccines (Basel). 2022 Apr 20;10(5):650. doi: 10.3390/vaccines10050650 (PMC9147477; doi:10.3390/vaccines10050650)
Supplement: Supplementary file 1 [file vaccines-10-00650-s001.zip › Supplementary Text S2-JE-CB-20-4-2022.pdf]

Supplementary Text S2:

Estimation of working time preservation (in days) at UKER due to a theoretical COVID-19 vaccination from the beginning of the pandemic based on the number of SARS-CoV-2 infections detected by PCR amongst UKER employees

Part 1. Loss of working time (in days) preventable by vaccination:

$a = c \times d \times b$

a = working time theoretically preventable by COVID-19 vaccination; b = average time of SARS-CoV-2 positivity; c = number of UKER staff members tested positive for SARS-CoV-2 infection by PCR since the start of the pandemic until April 2021; d = vaccine efficacy.

Values: b = 23 days; c = 408 (April 2021); d = 95% [9].

a (total) = 408 × 0.95 × 23 days = **8,915 days**

Part 2. Preventable loss of working time due to complete vaccination since the beginning of the pandemic, taking into account the inability to work due to vaccination:

$a' = c \times d \times b - e \times f$

e = estimated average time of incapacity to work for each completely vaccinated staff member; f = number of UKER staff.

Values: e = 0.72 days (according to our data; see main text). f = 8,560 (November 2020).

a' (total) = 408 × 0.95 × 23 days – 0.72 days × 8,560 = 8,915 – 6,163 days = **2,752 days**

Part 3. The number of prevented infections (g) at which the preservation of working time due to vaccination outweighs the incapacity to work due to vaccination is calculated as:

g = (e × f)/(d × b) = (0.72 days × 8,560)/(0.95 × 23 days) = **282**
